# Who are the users of synthetic DNA? Using metaphors to activate microorganisms at the center of synthetic biology

**DOI:** 10.1186/s40504-018-0080-3

**Published:** 2018-07-14

**Authors:** Erika Amethyst Szymanski

**Affiliations:** 1 Science, Technology, and Innovation Studies, University of Edinburgh, Edinburgh, UK; 2 Chisholm House, High School Yards, Edinburgh, EH1 1LZ UK

**Keywords:** Synthetic biology, Metaphors, Yeast, Multispecies research

## Abstract

Synthetic biology, a multidisciplinary field involving designing and building with DNA, often designs and builds in microorganisms. The role of these microorganisms tends to be understood through metaphors making the microbial cell like a machine and emphasizing its passivity: cells are described as platforms, chassis, and computers. Here, I point to the efficacy of such metaphors in enacting the microorganism as a particular kind of (non-)participant in the research process, and I suggest the utility of employing metaphors that make microorganisms a different kind of thing—active participants, contributors, and even collaborators in scientific research. This suggestion is worth making, I argue, because enabling the activity of the microorganism generates opportunities for learning from microorganisms in ways that may help explain currently unexplained phenomena in synthetic biology and suggest new experimental directions. Moreover, “activating the microorganism” reorients relationships between human scientists and nonhuman experimental participants away from control over nonhuman creatures and toward respect for and listening to them, generating conditions of possibility for exploring what responsible research means when humans try to be responsible toward and even with creatures across species boundaries.

## Introduction

Synthetic biology is a multidisciplinary field involving microbiologists and geneticists, engineers and computer scientists, designers, physicists, entrepreneurs, and an array of social scientists, artists, and civil society organizations who come to investigate and comment. Yet in this stew of workers, one group – huge, but tiny – is often present but rarely called out as such. Where are the microorganisms? They are often noted among the materials and tools, part of the necessary machinery for synthetic biologists’ work. Sitting in the laboratory, however, you might see something different: microorganisms are essential and active contributors. Synthetic biology is not only multidisciplinary, but multispecies.

From a science studies perspective, this is hardly surprising news. Actor-network theorists would have walked into synthetic biology labs with the working hypothesis that not just the yeast and bacteria but the PCR machines, the dissection microscope, the gene editing computer software, and the Nespresso coffee machine all contribute actively and necessarily to the production of *Science* papers and PhD students (for the classic example, see Latour and Woolgar 1979). Similarly, calling out the participation of the microorganism is unlikely to surprise scientists working in the lab, who structure their waking hours around the needs of their species of choice and whose life or death as scientists is tangled up in the life or death of their cells. And yet, in making sense of microorganisms’ scientific contributions, scientific discourse often makes them passive: “platforms,” “chassis” (as in the structural framework of an automobile), and “operating systems” (e.g. Cameron et al. 2014; Dietz and Panke 2010). These metaphors for what microbial cells are thought to be *like* shapes how scientists and other humans are encouraged to see microorganismal capacities and patterns how synthetic biologists iteratively remake microorganisms through their genetic construction work.

In this short paper, I observe that metaphors bring microorganisms into being in the synthetic biology lab as “experimental partners” with certain characteristics patterned by what they are described as being *like*, and of which certain expectations are therefore reasonable. I suggest that metaphors which invoke microorganisms as active participants in the DNA design process – in particular, seeing microorganisms as users of synthetic DNA – can allow microorganisms to do more, and potentially create new and productive opportunities for scientific learning in multispecies community. Experimentally, in both natural and social sciences, accounting for microbial participation affords new avenues for gathering richer information about microorganisms. In broader spheres, attending to and accounting for multiple forms of work, involving a range of creatures who contribute to the output of the lab, is a strategy for valuing that work and endeavoring to protect all organisms’ capacity to contribute (van Dooren et al. 2016). And in terms of conducting responsible research, first visualizing and then valuing the “stakes” of microorganisms is a step toward being responsible toward – and response-able *with* (Haraway 2008) – the myriad creatures whose wellbeing is entangled in scientific research rather than constituting responsibility in human terms alone. Metaphors which close down the roles nonhumans can occupy as living things in laboratories and other shared work-spaces, in short, limit the material-semiotic apparatus (Hayward 2010) through which we can learn and work together. More active metaphors for microorganisms can thus be seen as methods for doing multispecies research as well as for doing synthetic biology.

I root these suggestions in my experience with *Saccharomyces cerevisiae*’s participation in the synthetic yeast project. The synthetic yeast project, or *Saccharomyces cerevisiae* 2.0, is an international effort to design and build the first complete synthetic eukaryotic genome with which I spent 18 months as a resident social scientist, working alongside other members of a contributing laboratory, attending seminars, and participating in conferences and workshops. *S. cerevisiae*, common baker’s yeast as well as a crucial model organism across biological sub-fields, is one of the easiest organisms to see as an active participant and even a collaborator in shared human-yeast work. Evolutionary genetics research indicates that the varied *S. cerevisiae* who work with us are domesticated: they display recognizable genetic changes mirroring those of other domesticated organisms such that yeasts used for baking, brewing, winemaking, laboratory science, and industrial fermentation can be systematically distinguished from their wild counterparts found in oak forests in North America, Europe, and Asia (Fay and Benavides 2005). Using a different understanding of domestication, we might consider that humans and yeast have domesticated each other; we have become accustomed to living and working together and have mutually acclimated to shared environments in ways not wholly the product of human intention (Katz 2012; Larson and Fuller 2014).

Yeasts which have become part of laboratory science hold those positions because their behaviors make them easy to handle in experiments; preferred lab yeasts tend to grow as single cells rather than clumping together and have minimal nutritional requirements, for example (Langer 2016). While yeast strains used for genetic construction and various other scientific purposes have been carefully cultivated over innumerable generations for their amenability and particular skill in such work, an increasing variety of other microorganisms are also used with more or less success. The extent to which these efforts are successful – efforts to deliberately domesticate new microorganisms to human work – can be seen as reflecting the extent to which these microorganisms will participate in scientific labor. I hope that focusing on yeast, rather than eliding less systematically cooperative microbes, can be a point of entry into thinking through how varied microorganisms participate in DNA design work.

## Conceptual metaphors as synthetic biology construction tools

What is a microbial cell? Visible to the naked eye only through their activities en masse, human knowledge of microorganisms *as cells* is a product of scientific investigation transposing human vision to a different scale. What microbial cells become is a function of the tools used to visualize them. Some of those tools are material: microscopes, stains, tests for particular metabolic activities. Some are discursive. Like the slippery fish John Law and Marianne Lien bring into being through their experiment in empirical ontology at a Norwegian salmon farm, microbial cells can be said to be brought into being through performances, the “various practices that *do*” them (Law and Lien 2012, 366).

Amongst the Petri dishes, culture media, and PCR machines, metaphors are daily instruments in biology laboratories, and *language* practices are central to what yeast become. In Burke’s (1966) parlance, language constitutes an inescapable “terministic screen” through which language-users perceive and understand the world, “and any such screen necessarily directs the attention to one field rather than another” (50). Material and discursive tools alike are strategies for “doing” microorganisms, for bringing them into being through our interactions with them. Metaphors must be used to imagine invisible cells into being, to think about their capacities, to shape experiments and other operations that might be done with them, and thus to realize what the cell is.

In synthetic biology, microbial cells are often realized as “platforms” or “chassis,” the latter term referencing the metal frame of an automobile and invoking the cell as a basic underlying structure onto which genetic assemblies of interest are bolted (e.g. Adams, 2016; Cameron et al. 2014; Rabinow 2009). Synthetic biology is an umbrella term for an (in)famously heterogeneous range of research and biotechnology, some of which does and some of which does not involve microorganisms. At one extreme, “cell-free” synthetic biology involves building genetic circuits outside the supportive contexts of cellular environments, and “protocell” construction aims to design simplified cellular environments de novo from non-living components. At another extreme, some projects aim to redesign whole microbial genomes. In the middle, most of the “parts-based” work that has historically comprised the bulk of the field depends on microorganisms to serve as platforms, chassis, or operating systems to house and run custom-designed genetic circuitry.

In O’Malley et al. 2008 proposed a three-part classification for approaches to synthetic biology, describing projects as parts-based, whole-genome, or protocell work depending on relationships between the living cell and the parts being engineered. For the sake of focusing on microorganismal involvement, this article relies on a different analytic concerned not with the *approach to* design but the *context for* design: chassis-based synthetic biology, encompassing a large proportion of parts-based work as well as whole-genome engineering. I define chassis-based synthetic biology as work that involves inserting DNA constructs (typically designed in silico, that is, via computer-aided design) into living cells – the “chassis.” While cellular chassis may include mammalian cell lines and other cells derived from multicellular organisms, microorganisms – often *S. cerevisiae* and *E. coli*, these microbes long and uniquely domesticated to laboratory work *–* comprise the majority of chassis. Relating the scientific participation of mammalian cell lines bred for research and the participation of microorganisms is an interesting and worthwhile project, but one beyond the scope of this paper (see Landecker 2007 for beginnings into such a project from the mammalian cell culture side).

Investigating how microorganisms participate in chassis-based synthetic biology – as passive chassis, active users, or in any number of other roles – is at its heart a matter of investigating which *metaphors* are most useful or most appropriate for working well with microorganisms. When yeast cells are conceptualized as chassis, ways of working with those cells practice them or bring them into being as an inert structural component of a machine and make them more and more chassis-like. The discourse of chassis and platforms are terministic screens, directing attention to some of the cell’s properties and clouding over others.

Whether “chassis” is a metaphor, in addition to functioning as a terministic screen, depends on one’s definition of metaphor. Microbial cells are not “really” chassis – to use Lakoff and Johnson’s (1980) formulation – in the sense that cells are not encompassed by the traditional or primary use of the term. “Chassis” is a metaphor in that its application to cells involves applying a familiar framework to a new target outside its conventional use, encouraging readers to understand the new target through existing conceptual patterns and, in so doing, making the new target *like* some better-known thing. As Nietzsche (1994/1873) and many others have argued, however, *all* language can be seen as metaphorical insofar as we are continuously understanding new phenomena through frameworks developed in prior experience with other phenomena; we constantly and inevitably use language metaphorically when we make sense of new things by comparing them with things we have seen before via pre-existing conceptual schema. Calling any chair a chair is a metaphor in that the object is seen to be *like* some previously seen thing, a relationship that the language-user constructs and that imposes particular frameworks for understanding and working.

Because defining metaphor in Lakoff and Johnson’s (1980) more limited sense requires distinguishing between the core or typical meaning of a word and extended, unconventional applications, metaphors in this sense are discourse community-specific because core and extended meanings of words may vary across communities of speakers (Stelmach and Nerlich 2015). Discussing metaphors in science is complicated, therefore, by discursive differences amongst scientific communities and between scientific communities and more general public discourses. Consequently, rather than beginning with some understanding of what microorganisms “really are” and thereby indicating that they have a fixed and prior identity, a more useful starting position involves seeing all of these terms employed to describe what microorganisms may be as framing devices (Entman 1993) for encouraging different understandings of their target.

For examining how language practices act as framing devices to invoke their objects, rather than working to distinguish metaphor from ordinary language, a more useful distinction is between *conceptual metaphors* and *superficial metaphors*. “Juliet is the sun” is a *superficial metaphor*. Treating Juliet as though she is the sun – that is, bringing Juliet into being by doing things with Juliet, as Law and Lien (2012) “do” their salmon – is not useful, and is obviously not the point of the expression. In contrast, “the microbial cell is a chassis” is a *conceptual metaphor* in that the expression permits and encourages working with the microbial cell using operations that cohere with the idea that the cell is a chassis: functional modules may be loaded onto the cellular chassis, the basic structure of the cellular chassis constrains what kinds of parts can be loaded, the cellular chassis needs to physically house the modules, etc. A paradigmatic example of a conceptual metaphor in genetics is the “genetic code,” a concept which has permitted reading and writing, decoding and recoding DNA (e.g. Hellsten and Nerlich 2011; Kay 2000).

Conceptual metaphors must work with the materiality of their targets. Juliet does not emit the necessary electromagnetic radiation to heat the earth, allow plants to grow, or make looking directly at her impossible, while mounting knowledge about DNA has continued to cohere (well enough, at least) with the idea of the genetic code. But conceptual metaphors also constrain how the materiality of their targets is seen. If we do not begin with the prior assumption that microorganisms are too small, simple, or stupid to behave as independent living things, then it is easy to find evidence supporting their participation in DNA design and construction work.

## Conceptual metaphors as methods for multispecies research

As Buller (2015) observes of methods for multispecies research in general, metaphors are discursive methods for enacting (Mol 2002) microorganisms that alter the roles and capacities available to them in their human relations, in and outside of scientific laboratories. Buller, in reviewing methods for doing animal geographies, exhorts multispecies researchers to avoid generalizing nonhumans by relying too readily on ready-made categories such as species definitions or on social science–natural science divisions – recommendations with which attending to metaphor coheres by observing how microorganisms (in this case) are brought into being through specific microbe-human practices. Buller also recommends that researchers seek “approaches that do not rely upon wholly human representative accounts” (376), an ideal which seems not only unachievable but even undesirable when the concern is how humans and other creatures work together. Insofar as humans are limited to human perspectives and are invariably studying how nonhumans manifest in human worlds and consciousnesses, imagining that research methods could afford an escape from this limitation risks losing sight of the fundamental otherness of nonhuman experiences. Metaphors, as methods for “doing” microbes or other creatures, do not directly avoid relying on human accounts to understand nonhuman action; rather, they change the terms that establish what those creatures can become when they enter human conversation.

In human conversation, microorganisms may often be cast as “stupid” – or, more precisely, their actions may be seen as reflexive, or as operating within a very narrow range of possibilities tightly constrained by environmental stimuli (though perhaps chiefly when they are observed single cells in isolation rather than in communities capable of more complex behavior; see, for example, Shapiro 2007 on the communal intelligence of bacteria). Burke (1966), arguing that “‘observations’ are but implications of the particular terminology in terms of which the observations are made,” points to the utilitarian philosopher Jeremy Bentham’s conclusion that “all terms for mental states, sociopolitical relationships, and the like are necessarily ‘fictions,’ in the sense that we must express such concepts by the use of terms borrowed from the realm of the physical” (46). The same might be said of the microscopic needing to be described in terms of the macroscopic. Bentham concluded that the best course of action is to explicitly acknowledge such fictions as such because eliminating them entirely – Bentham’s ideal – is impossible. In cognate “fictions” for dealing with microbes, the problem and its resolution might be productively framed in another way. As creatures living in a macroscopic world, humans must use the tools available to us as macroorganisms to understand and make relatable microorganisms and others who do not inhabit these same worlds. Bentham’s problem of needing to tell “fictions” to describe nonphysical objects is of the same kind as the multispecies researcher who needs to use human relational or human performative terms to describe nonhumans. Anthropomorphism, as Jean Langford (2017) has recently pointed out, may be not a fault but an essential tool for coming to understand others who are necessarily outside the scope of our own experience.

Humans need conceptual metaphors to permit understanding microorganisms in terms of more familiar phenomena and making them visible and legible in the first place. Given this necessity, how do we decide which metaphors to use? Multispecies methods would advocate for beginning with openness about what microorganisms may be(come) rather than with assumptions on the basis of their differences from humans about what they are not. An example of such a beginning comes from Bastian et al (2017) “In conversation with...” project investigating the possibility of more-than-human participatory research, in which she asks “whether particular nonhumans have competencies that could support their involvement in PR [participatory research], and whether PR could develop methods that would support any such competencies” (28). Especially in light of increasing evidence that animals, plants, insects, and microorganisms can do far more to interact with their environments than has been previously realized, we might similarly look for how microorganisms *can* participate in research rather than assuming that they *cannot.* In what follows, I explore how attempting to follow that suggestion in working with the synthetic yeast project suggests a conceptual metaphor for the yeast – yeast as a user of synthetic DNA – that may in turn inform strategies for becoming more responsible (in the sense of navigating responsible research and innovation, e.g. Sliva et al. 2015; Stilgoe et al. 2013) and response-able (in the Harawavian sense of navigating ethical multispecies interactions; Greenhough and Roe 2010; Haraway 2008) with yeast in synthetic biology.

## Synthetic biology as a design discipline

Synthetic biology is often described as a design discipline involving designing and building with DNA (e.g. Agapakis 2014; Nguyen et al. 2016; Richardson et al. 2006; see also http://www.synbioproject.org/topics/synbio101/definition/). When employed as a verb, “design” invokes a designer – someone who designs – and a product created by the designer. “Design” also implicates a user – someone who makes use of the designer’s product. In synthetic biology, “DNA designers” have been characterized as interdisciplinary scientist-engineers, members of a boundary-crossing field where engineering principles meet biological systems. But who are synthetic biology’s users?

Synthetic biology’s would-be prophets are inclined to respond: everyone. Visions of the field’s future often position synthetic biology-driven products in consumer niches across research and industry, used by private individuals at home as well as at work. Some synthetic biology products have indeed entered industrial production, where both the industry itself and the end user of the industrial product can be seen as users of synthetic biology. In the case of the “synthetic-natural” vanillin produced in engineered yeast by the Swiss biotech company Evolva, for example, a chain of users can be drawn from: scientists, using purchased DNA to make a synthetic construct → to Evolva, using synthetic biology designs to make a product → to Evolva’s corporate customers, using synthetic vanillin to make sweet-smelling hand soap (for example) → to someone who purchases the vanilla-scented hand soap, using the soap as part of a daily hygiene routine. This and other such chains miss an important initial step. Before the scientist can successfully generate a synthetic biology product, the microbial cells involved in the design process must first be able to use synthetic DNA to support or permit normal cell growth and reproduction and, simultaneously, to perform the function desired by the human scientist. Microorganisms are the initial and most crucial users, for if they do not succeed no would-be user further down the chain can even try. Describing microorganisms as users does not exclude or downplay the importance of attending to end-users of synthetic biology applications further downstream, be they domestic hand-washers, biologists studying cell division, public health workers using an arsenic biosensor to test well water, or other synthetic biologists. Rather, microorganismal use is a necessary precursor to all subsequent uses.

Changing conceptual metaphors is one strategy for reframing research such that participants are not a priori excluded on the basis of their not being human, or because their form of participation does not look like what we expect from (specific categories of) human participants. Reframing research to permit participation by nonhuman creatures changes the kinds of experimental questions that can be reasonably asked, the capacities the microorganism might be expected to have, and thus the kinds of work that can be done with them. Through this screen, what might user-centered design mean in microbial synthetic biology? How could microbes be positioned at the center of a synthetic biology design process? How do humans design for, and even design with microorganisms? Such questions should encourage more considered attention to the unique properties of microorganisms – in and outside of synthetic biology – as living creatures which, by being so unlike macroorganisms whose agency is easier to conceptualize, are more easily treated like machines. Following Buller’s and other’s suggestion, that attention might look both at “the performance of routine practice” and “to eventful and troubling interruptions” where non-human activity “interrupts” those routines (Buller 2015, 337). In what follows, I use the synthetic yeast project to exemplify how understanding microorganisms as users of synthetic DNA, coheres with the material properties of how microorganisms are practiced in the lab and thus can be applied as a conceptual metaphor and not just a superficial one, that is, as a constructive scientific tool for structuring modes of thinking and working.

## Yeast as user in the synthetic yeast project

*Saccharomyces cerevisiae* 2.0, Sc2.0, or the synthetic yeast project, is an international project involving 11 laboratories collaborating to construct the first complete and comprehensively redesigned eukaryotic genome entirely from laboratory synthesized DNA. Yeast’s selection as the object of this first foray into whole-eukaryote genome construction is testimony to the established habit in genetics and genomics of treating yeast as the simplest eukaryotic organism (see Langer 2016). The choice also reflects how yeast’s inclination to cooperate makes constructing the yeast genome more achievable than the same sort of project would be for any other eukaryote. Yeast provides the genetic material serving as the template for the genome being constructed, but also participates in far more active ways.

*S. cerevisiae* are central actors in genetic assembly work, not just in the synthetic yeast project but across synthetic biology and related biotechnologies more generally, thanks to yeast’s extraordinary expertise in homologous recombination. For yeast cells, homologous recombination is a means of repairing breaks and other snafus in DNA replication (Eckert-Boulet et al. 2011); for human scientists, the process becomes a means of assembling DNA segments by matching and integrating across overlapping sequences (Symington 2006). Other human-designed strategies for assembling DNA exist – Golden Gate and Gibson assembly, for example – but homologous recombination in yeast often succeeds where these strategies have failed. Scientists do not yet comprehensively understand how homologous recombination works and cannot replicate it “ex yeasto”, and so routinely rely on yeast’s expertise.

Yeast, consequently, are responsible for a massive and essential part of genome construction work in the synthetic yeast project. Short segments (the length varies across contributing laboratories) or “chunks” of purchased DNA (DNA synthesis is a competitive private industry) are assembled into longer “megachunks” using restriction enzyme sites to create complementary sticky ends permitting adjacent segments to link together in the correct order in vitro, a method preferred for its speed (Richardson et al. 2017; Mitchell et al. 2017). Thereafter, however, replacing the native genome of a living yeast cell with chunk after chunk of the synthetic genome is the work of the yeast. Sections of synthetic DNA are transformed into live yeast cells using their inducible inclination to take up DNA from their environments. The yeast, by homologous recombination, then replace sections of their native chromosomes with the synthetic homologues. The human scientists wait. Scientists can then select for “correct” cells with the desired constructs by growing the population of cells which have been asked to perform this DNA assembly work in Petri dishes on selective media, on which only cells which have held on to the most recently added synthetic megachunk will be able to grow. “Failure” cells die or are unable to reproduce; either way, failures become invisible. Sampling DNA from colonies which grow from such correct cells – visible to the naked eye on the surface of solid media – and examining that DNA via PCR reactions with probes specific to the new megachunk usually indicates that visible colonies are the progeny of yeast cells which have successfully done the work asked of them.

Interruptions in this routine process – instances in which megachunks do not integrate smoothly, when few colonies grow, when they grow too slowly for the normal pace of scientific work, or when the smooth surface of agar-solidified growth media is completely blank – are instances in which we might look for the action and frame the *enaction* of the non-human partner, instances in which scientists have opportunities to listen for yeast to say something about what it is being asked to become. Yeast cells’ response to synthetic sequences is the first measure of whether a redesigned sequence is a success or a failure. Even if a physical genetic construct is assembled to perfectly match the in silico plans for that construct, that “perfect” construct may not constitute a success if inserting it into a cell does not elicit the desired response. Examples of genetic constructs assembled according to their design plans that nevertheless fail to meet design goals are legion in synthetic biology. Conversely, one synthetic yeast laboratory’s efforts to build a “perfect” version of their assigned synthetic chromosome, one identical to the design plan set out in the in silico (digital) blueprint for the genome (Xie et al. 2017), might necessitate correcting small mutations that do not appear to impede cell function, or that even produce a version of the chromosome that works better for the yeast cell than the version originally designed by the human scientists and their computer algorithm partners.

Yeast cells can, thus, be seen as users of synthetic DNA. When a segment of redesigned DNA is inserted or “transformed” into yeast, the first thing that must happen is that the synthetic sequence must align with matching sequences in genomic DNA that indicate where the human scientist plans for it to integrate. Having incorporated the new segment in its correct position, the yeast must then be able to use the new synthetic sequence to support cell function. The scientist’s changes may make the yeast cell unable to interpret the synthetic sequence, or the function of the sequence once interpreted may fail to perform or interfere with necessary cell tasks. If the yeast is unable to use the redesigned sequence, it may either die, fail to grow as robustly as necessary to continue with additional experiments, or fail to perform the behavior that the redesigned sequence was supposed to elicit. Put differently, the yeast may indicate its inability or unwillingness to work with the technology the scientist has designed, refusing to show up for additional work or expiring because its basic needs aren’t being met. In all such cases, the synthetic sequence will have failed and must be redesigned, or the expectations of the scientist will need to change to accommodate the yeast’s response.

Myriad other framings are possible. Following Woolgar (1990) to address synthetic DNA as a text and therefore as interpretively flexible (see also Fish 1980), synthetic chromosome construction might be recast as a process of negotiating textual meaning amongst a multispecies community of readers. Microbes might be guests at the invitation of the scientists, lodged and fed while being asked for their invaluable help. They might be captives, held and genetically modified in ways that often result in their death. While any number of metaphors might invite us to see something new about synthetic biology, a particular utility of seeing microbial cells as users lies in the possibilities it invites for applying user-centered design principles to think about how microbes actively participate in the design process. When microbes are positioned as users, and the usability of DNA by those microbes is positioned as the foundation of all successful synthetic biology design, ensuring usability by accounting for users’ needs becomes paramount.

## Accounting for microbial needs, valuing microbial contributions

User-centered design (UCD), as a broad category of design approaches, proposes that good design must account for users’ needs and preferences rather than forcing users to adapt to technologies structured around designers’ ideals. Cooperative or participatory approaches to UCD aim to reduce or eliminate the hierarchy between users and designers, recognizing the knowledge, expertise, and interests of both groups (for relevant discussions, see Binder et al. 2015; Jönsson and Lenskjold 2014; Salvo 2001). Many differently theorized forms of user-centered and participatory design exist, some of which rely on connections to actor-network theory through “participation” to re-conceptualize design projects as heterogeneous assemblies, re-focusing entirely from thing-as-object to thinging-as-process (Telier 2011). Yet at the heart of all of these approaches is a reconceptualization of expertise to make possible, make visible, and make valuable the knowledge of those outside of traditionally conceived expert groups. UCD proposes that the most successful means of doing design assumes that designers do not have access to absolute knowledge which retains its truth status independent of context, and that designers and other experts therefore have something to learn from those – potentially including non-humans – with different knowledges.

When design and knowledge relevant to design are no longer seen as the sole provenance of traditional experts and may be held by others – including nonhuman others, potentially – UCD becomes congruent with actor-network theory and participatory design becomes about doing more-than-human worlds. Highlighting that congruence, Binder and coauthors Binder et al. (2015) present “participation” as a process of “drawing things together” (quoting Latour), constantly in process, in which the shifting assemblage of sociomaterial things makes clear that humans are not the only participants. Reading participatory design as a matter of “thinging as socio-material assemblies that evolve over time,” they suggest, “leads us on to fertile ground for experimentation that goes beyond the taken-for-granted wisdom that the user is king, and that human-centredness is a solid ground” (152). They conclude that invitation must replace representation such that design becomes a tool for doing democracy.

Notwithstanding possibilities for envisioning multispecies democracy – or, equally, arguing against it (e.g. Eckersley 1995) – a more immediate consequence of microorganismal participation in synthetic biology is the capacity for growth and surprise amongst labmates. By making microorganisms tools or machines, microorganisms are imagined as being comprised of constituent parts of their desirable capacities plus troublesome “complexity” that needs to be engineered away in the service of more tightly controlled and thus more successful engineering (e.g. Cobb et al. 2013; see also Keller 2005). By inviting microorganisms to be organisms with different knowledges and capacities than scientists, even while synthetic biology remains an unquestionably human endeavor, microorganisms are allowed the possibility of response. In listening for those responses, scientists retain the possibility of being surprised by, learning from, and making use of capacities which they do not own, do not control, and do not need to know how to perform. Enacting microorganisms as mechanical structures, in contrast, limits scientists to seeing what they already know.

The way scientists who work with yeast conceptualize what yeast is capable of has material consequences. From perspectives that see reality as being simultaneously material and “shaped by modes of understanding and engagement,” as van Dooren et al. (2016) professes on behalf of multispecies studies, “ways of knowing and understanding have profound consequences: they shape worlds” (12). In the synthetic yeast lab, the texture (Lien and Law 2012) of that action is especially tight. Ways of understanding yeast guide ways of remaking the yeast genome as well as ways of bringing yeast into being as a thing, organism, set of capacities, or participant in the lab. Even without accepting any reductionist equation of the genome with the organism, we can see synthetic yeast construction workers building a new version of the organism, shaping yeast according to the pattern of their imaginations.

What is at stake thus includes the nature – or more aptly, the natureculture, especially for this domesticated organism – of yeast as well as of humans, the shape of the world they mutually inhabit, and their capacity to learn together. Synthetic biology is remaking microorganisms to be more like the sources of the metaphors applied to them. In so doing, synthetic biology is remaking humans-in-multispecies-relations, creating the possibility of exercising human dominion over the earth by making humans designers and programmers of living things, directors *over* rather than learners *with*.

As Landecker (2016) supports through her “biology of history” of antibiotics, material effects of scientific understandings of microorganisms is by no means a new phenomenon; on the contrary, a microorganism as an object of study “has the human history of explanation and intervention within it” (37) – a point, as Landecker notes, increasingly articulated by microbiologists themselves. Chassis-based synthetic biology, as a group of cases of microbial material-semiotic embodiment, does not warrant attention because such activity is *new*, but because it is still happening. And indeed, synthetic biologists often introduce their work by observing that humans have been designing living things since the first days of agriculture and domestication. Nevertheless, two important differences in contemporary synthetic biology warrant marking a discontinuity in this lineage. One: the locus of human activity in synthetic biology is understood to be the central “operating system” of the organism. Even though biological knowledge challenges any notion of reducing the organism simply to the genome, genomes are still widely perceived as the driving force or “conductor” of cells, and synthetic biology relies on behaving as if DNA is sufficient to program organismal behavior. Two: the metaphors applied to microorganisms in synthetic biology enable different modes of acting and different discursive frameworks for conceptualizing action in the reciprocating material-discursive tangles shaping the world as we know it.

In the synthetic biology lab, these political matters become practical questions: how to work with microorganisms? What words to use to shape what microorganisms can do? Metaphors are very practical tools in making some facets of the organism count and making others elements to be unknowingly ignored, casually discarded, or deliberately engineered away.

Experiments in involving microorganisms in participatory design might be informed by similar experiments with nonhuman macroorganisms. Despret (2004) shows us how historic cases of multispecies research, often framed as polluted by inadvertent bodily communication, might instead be understood as instances of multispecies learning within which being an embodied researcher and “learning how to address the creatures being studied is not the *result* of scientific theoretical understanding, it is the *condition* of this understanding” (131). Bastian, Jones, Moore, and Roe (2016) have recently explored the possibility of participatory research with non-human creatures, placing themselves and their colleagues “in conversation with” domestic dogs, bees, trees, and water investigating the possibilities afforded by working “with particular animals, insects, plant and elements specifically as *research partners*, rather than say as subjects of experiments” (20). While the team chose these partners on the basis of their own expertise and research network, the point was less to focus on these entities specifically than on the challenge of working with partners who were not human. In their experience and the experience of other contributors to their edited volume on more-than-human participatory research, the most productive experiments – those yielding experimental findings which could be taken forward for additional multispecies research – were those in which the nonhuman was intrinsic to the process.

## Communicating with microbial collaborators: operationalizing “with”

An important question then becomes: how do scientists communicate with yeast? A majority of interspecies communications in synthetic biology labs happens by way of growth rate. Cells communicate their satisfaction or dissatisfaction with synthetic DNA by growing at a normal rate, by growing more slowly, by refusing to grow at all, or by dying. Communication may also occur via such signals as color when an output of a synthetic sequence is linked to the production of a colorful molecule that enables yeast to communicate with yeastworkers chemico-visually, even without technologically augmenting the scientist’s somewhat limited sensory capacities. Fifteen years ago, Jasper Rine (2006), a yeast biologist at the University of California, Berkeley, suggested that microbiologists need to be more nuanced in their modes of listening in his introduction to *Landmark Papers in Yeast Biology*:

Too often, we dismiss a mutant’s phenotype as being slightly sick or slow growing. At our present level of sophistication, phenotype is what we observe after the cell has exhausted its ability to compensate for the loss of some gene. If we can go beyond our present and often superficial phenotyping and develop better ways of asking a cell, ‘Where does it hurt?,’ we will create studies that will be the landmarks of biology and not just of our field. (7).

From the position that yeast have other valuable things to say, we might also go beyond asking a cell where it hurts and think about eliciting responses to more nuanced questions. As microbiologists increasingly study microorganisms in complex communities rather than as artificially isolated, genetically identical populations, they report finding that those communities communicate in sophisticated ways. J.A. Shapiro’s (2007) assertion that “bacteria are small but not stupid,” for example, follows from four decades of experience in bacterial genetics during which his studies of complex and highly coordinated communication amongst bacterial cells led him to conclude that “there are no units, only interactive systems” (816). While the individual bacterial cell may appear from a human scientist’s vantage point to have little scope for complex behavior, observing how bacteria typically live in community and interact with their environs in ways beyond “the organism” suggests a different conclusion. Shapiro’s suggestion is about attending more to what microorganisms *do* and to how they function in multispecies environments than to defining what microorganisms *are* through concepts of “individual” and “agency” inevitably structured through *human* experience and expectations.

Work in synthetic biology, guided by central principles of modularity and decoupling, has tended to move in the opposite direction. Aiming to make biology modular, synthetic biologists have worked toward creating units of biological function *qua* DNA that can be standardized, black boxed, and recombined to create novel composite functions even with little knowledge of biology (e.g. Shetty et al. 2008), and with any need to attend to the “interactive system” either built into the part itself or engineered away. An extreme example of one approach to microbiomodular design can be found in Douglas Densmore’s Cross-disciplinary Integration of Design Automation Research (CIDAR) group at Boston University, where a formal design language modeled after similar languages in computer programming is being developed to operationalize microbial cells as programmable design spaces (Bhatia et al. 2017). While Shapiro (2007), voicing a position more common amongst microbiologists, allows that comparing microorganisms to computers can be useful “to think concretely and scientifically about complex information processing,” he warns that

we should not allow the electronic computation metaphor to become another intellectual straitjacket. Our digital electronic computing systems are far simpler than the distributed analog processors in living cells. The take-home lesson of more than a half a century of molecular microbiology is to recognize that bacterial information processing is far more powerful than human technology. (816)

His lesson is that we should not employ metaphors to make microbes so much simpler than they can be and, in so doing, to limit both their abilities and ours to what we currently see. In contrast, metaphors that allow for microorganisms’ active participation generate possibilities for learning in far more detail what microorganisms can do and what they can become.

## Conclusion

### Being responsible with metaphor

Inviting the active participation of microorganisms in DNA design might have very real and productive consequences, for synthetic biology, for multispecies studies, and for the points of contact between these forms of research. “Activating microorganisms” by structuring their roles and abilities through different conceptual metaphors is therefore a worthwhile experiment for at least three reasons. First, conceptualizing microorganisms as users allows for more complete descriptions of the kinds of work happening in synthetic biology. Allowing for more active participation of microorganisms enables explaining laboratory observations through more nuanced means of gathering information from yeast. Seeing yeast as active participants with something to say may, in this way, guide more effective experimental design by providing more and more nuanced opportunities to learn from microbial action. Building synthetic chromosomes can be seen as a matter of human scientists and yeast learning how to do this work, and learning together, with the abilities of each partner altering the landscape of (inter)action for the others.

Second, positioning microorganisms as users draws attention to ways in which synthetic biology remakes the nature of being human through changing the nature of multispecies relationships through which being human is constituted (Tsing 2012). When microorganisms become factories, chassis, or computers, able to be designed and programmed, humans become designers and programmers of living things. Reframing the participation of the microorganism reorients these relationships between human scientists and nonhuman experimental participants away from control over nonhuman creatures and toward mutual respect and listening, thereby generating conditions of possibility for exploring what responsible research means when humans try to be responsible toward and even with creatures across species boundaries.

Third, and relatedly, discursively enabling microbial participation should provoke reconsidering responsible research in a multispecies light and, in so doing, attending to how synthetic biology operates with and has stakeholders amongst complex, diverse communities of humans and non-humans. Framing responsible research as a multispecies activity expands – and, indeed, improves the basic framework for conceptualizing synthetic biology’s overarching goals for synthetic biology: to create better futures (Ginsberg 2017). These futures can never be just about designers and designers’ needs, or indeed about humans and humans’ needs. Future worlds will be inhabited by *everyone*, broadly defined and including many varieties of living creatures. Work toward “better” futures must take into account the needs and goods of yeast, bacteria, scientists, other “species” of humans, and all of the others who will inhabit the future and participate in creating it.

In invoking the presence, valuable contributions, and even “stakes” of nonhuman research participants, I in no way mean to elude the gross and inevitable inequalities amongst scientists and their microbial and other nonhuman research participants that have been discussed across multispecies animal research. Investigating whether and how similarly structured inequalities might matter for multispecies microbial research is, perhaps, part of the agenda for multispecies responsible research that takes microbial life into account. As Stengers uses the term “obligate,” and Despret and Meuret (2016) after her, discursively structuring scientific research to allow for such questions *obligates* us to ask such questions, and to pay attention to how research interacts with others, broadly conceived. To the extent that we are always invariably living and working in multispecies community – thus, always – the starting point for conducting research responsibly to produce future worlds that serve *everyone* well, human and not, must be acknowledging the presence, participation, and worth of the nonhumans.

## Abbreviation

UCD: user-centered design
